# “This Is Me” an Awareness-Raising and Anti-Stigma Program for Undergraduate Nursing Students: A Pre-Post Intervention Study

**DOI:** 10.3390/nursrep14040216

**Published:** 2024-10-12

**Authors:** Olga Valentim, Tânia Correia, Lídia Moutinho, Paulo Seabra, Ana Querido, Carlos Laranjeira

**Affiliations:** 1 Nursing School of Lisbon (ESEL), Av. Prof. Egas Moniz, 1600-096 Lisboa, Portugal; lmoutinho@esel.pt (L.M.); pauloseabra@esel.pt (P.S.); 2 Nursing Research, Innovation and Development Centre of Lisbon (CIDNUR), Av. Prof. Egas Moniz, 1600-096 Lisboa, Portugal; 3 Center for Health Technology and Services Research (CINTESIS@RISE), Nursing School of Porto (ESEP), 4200-450 Porto, Portugal; tcorreia@essv.ipv.pt (T.C.); ana.querido@ipleiria.pt (A.Q.); 4 School of Health Sciences, Polytechnic Institute of Viseu, 3500-843 Viseu, Portugal; 5 School of Health Sciences, Polytechnic University of Leiria, Campus 2, Morro do Lena, Alto do Vieiro, Apartado 4137, 2411-901 Leiria, Portugal; 6 Centre for Innovative Care and Health Technology (ciTechCare), Polytechnic University of Leiria, Campus 5, Rua das Olhalvas, 2414-016 Leiria, Portugal; 7 Comprehensive Health Research Centre (CHRC), University of Évora, 7000-801 Évora, Portugal

**Keywords:** social stigma, disability discrimination, mental disorders, nursing students, education, literacy

## Abstract

Background: Stigma education for nursing students has focused solely on stigma reduction, with studies showing temporary improvements in attitudes. However, nursing education research should also emphasize the importance of critical reflection and self-reflection to enhance attitudes, beliefs, topic comprehension, and learning satisfaction. This study aimed to evaluate the effectiveness of the “This is me” intervention regarding knowledge, attitudes, and communication skills of senior undergraduate nursing students in responding to mental illness-related stigma. Methods: This study employed a psychoeducational intervention for reducing mental illness stigma, using a questionnaire survey to assess pre- and post-intervention effects, with 37 eligible nursing students undergoing clinical training in psychiatric services between 16 May and 15 July 2022. Instruments included sociodemographic and health questions, the MICA-4 scale to evaluate students’ attitudes toward mental illness, the MAKS to measure mental health knowledge, the Empathy Scale (JSPE-S), the Intergroup Anxiety Scale (SS-12), and the Attribution Questionnaire (AQ-27). Results: Most students were female (73.0%) and single (70.3%), with a mean age of around 29 years. After implementing the psychoeducational program, there was a statistically significant increase in overall stigma-related knowledge (MAKS: Z = −1.99, *p* < 0.05), a decrease in intergroup anxiety (IAS: Z = −3.42, *p* < 0.05), and reductions in the perceptions of patients as dangerous (AQ27—Dangerousness: Z = −2.399, *p* < 0.05) and fear (AQ27—Fear: Z = −2.415, *p* < 0.05). Additionally, there was an improvement in empathy, specifically in Perspective Taking (JSPE: Z = −2.555, *p* < 0.05). Conclusions: This program may contribute to mental health literacy related to stigma, positively impacting therapeutic relationships and communication with people with mental illness and resulting in more effective care practices.

## 1. Introduction

One of the primary targets of the Comprehensive Mental Health Action Plan 2013–2030 updated by the World Health Organization (WHO) is to mitigate the stigmatization experienced by individuals with mental disease [1]. According to the 2020 WHO Mental Health Atlas [2], about 52% of the preventive and promotion programs reported by WHO Member States were focused on enhancing mental health literacy or addressing stigma. These findings significantly emphasize initiatives to increase the public understanding of mental health and reduce societal biases. There is a growing number of global projects about mental health promotion and prevention: “18% were aimed at improving mental health awareness or combating stigma, 17% were school-based mental health prevention and promotion programs, and 15% were aimed at suicide prevention” [2] (p. 1).

A recent meta-analysis on the effectiveness of anti-stigma interventions for young people from 14 countries distributed in Europe, North America, Australia, and Asia suggests that education-based interventions show relatively more significant effects than other types of interventions [3]. Education-only interventions provide accurate mental health information through instructional sessions and seminars intending to question and dispel mental health misconceptions [4]. Education plus contact interventions seek to reduce anxiety and foster empathy by facilitating interactions between individuals and others who have mental health disorders [5,6]. The delivery modalities should be interactive, requiring participants to actively engage with facilitators and peers (e.g., via discussions), rather than simply absorbing material passively from facilitators. These evidence-based principles for stigma reduction consistently demonstrate good outcomes in terms of increased knowledge and improved attitudes via interventions at intrapersonal and interpersonal levels [5]. Although there was an increase in research articles on the stigma of nursing students and professionals towards people with mental disorders, few were aimed at designing and measuring the effectiveness of interventions to reduce it [7,8]. Most of these studies developed interventions that mitigate stigma (i.e., stereotypes [cognitive domain], prejudice [affective domain], and discrimination [behavioral domain]) [9] by promoting intergroup interactions between those with a stigmatized attribute, namely mental illness, and those without such attributes [10,11].

There is a need for more research into the most effective strategies, their target populations, and the specific contexts in which they are most successful. A comprehensive compilation of evaluations in the literature on the topic of Stigma Research and Global Health revealed significant deficiencies in the scope of research conducted and the overall quality of the studies [12,13]. For instance, one notable limitation is the way treatments targeting stigma are often developed, executed, and assessed, primarily concentrating on a singular context or healthcare system level, and are confined to a certain realm of stigma [12]. Another limitation is the absence of systematic methodologies for culturally and contextually adapting anti-stigma interventions deployed in various global contexts [14]. In the same vein, another limitation in the field of stigma reduction is the absence of behavioral tools and reliable measuring methods that are appropriately tailored to certain situations. This constraint hinders the ability to provide empirical data about the effectiveness of interventions in different settings.

Stigma is a significant obstacle in the realm of mental illness, impeding efforts related to prevention, treatment, and rehabilitation. Nursing students and professionals have the perception that people with mental diseases are hazardous, unpredictable, and emotionally unstable. Consequently, they often feel emotions such as dread, guilt, and animosity towards patients who are diagnosed with psychiatric disorders [8,15,16]. Healthcare workers exhibit a reduced willingness to provide care for these patients or administer unbiased treatment due to the traditional link with risk [17]. In this sense, the framework of mental illness stigma was used specifically to encompass the prevailing means by which individuals encounter stigma, to tackle fundamental elements of mental illness stigma that apply to a wide spectrum of mental disorders, and to distinguish between mechanisms that are most significant for individuals without mental illness—stereotypes, prejudice, and discrimination—and those that are most pertinent for individuals with mental illness—experienced, anticipated, and internalized stigma [18].

Research addressing undergraduate nursing students’ stigma concerning mental illness highlighted negative feelings and attitudes towards mental health consumers and unpreparedness for mental health clinical placement. Nevertheless, recent studies reveal the positive effect of nursing educational programs incorporating anti-stigma programs and mental health clinical placements in reducing social distance, improving recovery attitudes, and providing a prime opportunity to attract students into mental health nursing [19,20]. Anti-stigma programs were recently systematically reviewed by Guerrero et al. [21] who examined “the theory base, content, delivery, and evidence for the feasibility and effectiveness of interventions for healthcare professionals or healthcare students that aim to equip them with knowledge and skills to aid their patients to mitigate stigmatization and its health impacts” (p. 1). Thus far, stigma education for nursing students has only addressed stigma reduction, with studies demonstrating transient improvements in attitude [7,8]. These efforts have paid little attention to nursing education research, which emphasizes the importance of critical reflection and self-reflection as tools for enhancing attitudes, beliefs, topic comprehension, and learning satisfaction. Critical reflection facilitates students’ comprehension and evaluation of clinical scenarios based on evidence, hence augmenting their proficiency in problem-solving [22,23]. Additionally, it offers a deeper understanding of one’s own performance through metacognition, diminishes the likelihood of adverse events, and enhances communication with both patients and coworkers [24]. Likewise, the students’ cognitive empathy scores increased when they engaged in writing comments on their learning experiences [25].

The WHO, in its Comprehensive Mental Health Action Plan 2013–2020, strongly recommends the establishment of initiatives aimed at ameliorating stigma and psychiatric concerns in educational settings [1]. Recent evaluations of anti-stigma interventions revealed that knowledge, awareness, and attitudes regarding mental health disorders can improve in the short term [26,27]. Research examining the effects over an extended time (more than four weeks) indicated that although there was evidence of improved knowledge and attitude, these advantages failed to materialize in enhanced behavioral outcomes [27]. However, to date, few studies have been conducted in Portugal that examine the impact of an awareness-raising and anti-stigma intervention [28,29], aimed at reducing stigmatizing beliefs and attitudes among nursing students.

In this vein, this study aims to evaluate the effectiveness of the “This is me” intervention in addressing mental illness-related stigma among senior undergraduate nursing students in terms of knowledge, attitudes, and communication skills. We hope this research will provide insights into the potential effects of a contact-based intervention and a combined education program in our community and among populations of comparable cultural backgrounds.

## 2. Materials and Methods

### 2.1. Study Design

A pre-post study design was adopted, without including a control group. This study employed the anti-stigma intervention with a descriptive approach, using a questionnaire survey for pre- and post-intervention effects. A pre-post design without a control group is a type of study design in which data are collected on two different occasions: before and after an intervention, without a control group. In this type of study, participants serve as their own control, allowing changes to be assessed over time within the same group [30].

We obtained authorization from the school administration, via letter, for the program’s implementation and submitted the research protocol to the ethics committee for review and approval. Additionally, we negotiated the use of school resources to implement the program, including classrooms, computers, and projectors. We informed students about the program during theoretical classes in Mental Health and Psychiatry, encouraging their participation, and scheduled sessions according to the availability of students and school resources. We maintained clear and regular communication between students and teachers to address their needs, expectations, and concerns.

### 2.2. Participants and Recruitment

The participants were recruited from students at a private nursing school located in the Lisbon area. They were invited through the university’s official communication channels. Due to the exploratory nature of this study, a formal sample size calculation was not performed. Recruitment was based on student availability during the designated period, and the sample was considered appropriate for this pilot study. A convenience sampling technique was employed based on the following inclusion criteria: (a) being an adult; and (b) having completed the theoretical component of the Mental Health and Psychiatric Nursing Curriculum (3rd year course). Third-year students who were not enrolled in the mental health nursing course were excluded as well as students enrolled in mobility programs and those lacking proficiency in the Portuguese language.

**Figure 1:**
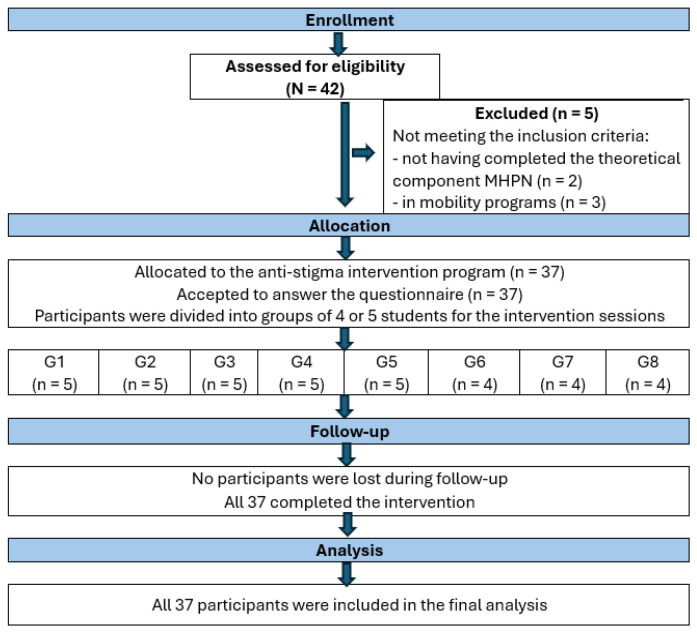
CONSORT (Consolidated Standards of Reporting Trials) chart for the interventional study.

Of the total eligible participants (N = 42) from a single institution, 37 students were recruited to participate in the anti-stigma program for nine weeks (between 16 May and 15 July 2022, totaling 250 h) while undertaking a clinical nursing internship in mental health at inpatient services in psychiatric hospitals in the Lisbon and Tagus Valley area. Figure 1 presents a CONSORT flow diagram that illustrates the progression of participants from enrollment to this study’s completion. Recruitment occurred during a period of pandemic-related restrictions, resulting in some limitations in available services for students. This also affected the capacity to recruit students for the present study.

### 2.3. Procedure

In the first phase, the authors interviewed third-year nursing students and collected data on sociodemographic characteristics (gender, age, marital status, nationality) and health (whether they suffered from mental illness or had any contact with a family member with mental illness), through an online questionnaire. Students who did not meet the inclusion criteria were excluded.

In phase two, face-to-face intervention sessions were scheduled according to participants’ availability. The intervention program involved 37 students, who were grouped into work teams of 4–5 members each.

Two faculty nurses carried out and supervised all program phases. The program’s first session took place 24 h after completing the questionnaire. The pre and post-test surveys were largely identical [latest on average 20 min to complete], except the pre-test questionnaire requested sociodemographic and health information. Additionally, the post-test questionnaire included a question to assess students’ opinions about the program and the degree of acceptability.

Ensuring a high retention rate of participants is crucial for maintaining the validity and credibility of interventional research [31]. A solid rapport between investigators and participants is crucial to maintain their involvement. Furthermore, providing individualized attention, which involves actively listening to each participant’s concerns and facilitating communication, fosters participant retention.

### 2.4. Measures

The assessment instrument is divided into sociodemographic form (age [in years], gender [male, female, other], marital status [single, married/cohabitating, divorced/separated/widowed], nationality) and health-related information (personal history of mental illness and family history of mental illness), along with scales validated for the Portuguese population. Lastly, there was an open question where participants were asked how the program contributed to self-awareness about stigma in mental health.

#### 2.4.1. Primary Outcome (Attitudes towards Mental Illness)

1. The Mental Illness Clinician’s Attitudes Scale (MICA-4)—Developed in King’s College London [32,33], this scale was translated and validated by Tomás et al. [34]. This tool allows for the assessment of attitudes towards mental illness among students in the healthcare field, such as nursing, pharmaceutical sciences, and psychology, aiming to understand the levels of associated stigma. Comprising 16 items on a Likert scale, with scores ranging from 1 (Strongly Agree) to 6 (Strongly Disagree), the score is calculated by summing the values corresponding to each response, after recording items formulated in reverse (1, 2, 4, 5, 6, 7, 8, 13, 14, and 15). The total score can vary between 16 and 96 points, with a lower total score indicating fewer stigmatizing attitudes towards individuals with mental illness. In the present study, this scale demonstrated reasonable internal consistency (Cronbach’s alpha of 0.65), which is slightly lower than Cronbach’s alpha of the original scale α = 0.72 [32].

#### 2.4.2. Secondary Outcomes (Knowledge About Mental Health; Empathetic Behavior; Intergroup Anxiety; Social Stigma and Stigmatizing Attitudes)

1. Mental Health Knowledge Scale (MAKS, originally developed by Evans-Lacko et al. [35]; Portuguese version by Camarneiro [36])—This instrument was developed as an indicator of knowledge about mental health and is divided into two distinct sections. The first part includes six statements that explore knowledge of factors associated with mental health stigma: help-seeking, recognition, support, employment, treatment, and recovery. The second part includes six statements referring to the classification of various conditions as mental illness. Each statement is evaluated on a Likert scale from 1 to 5, where “strongly disagree” corresponds to 1 and “strongly agree” corresponds to 5. Questions 6, 8, and 12 have reversed scores. The final score is obtained by the total sum of points, ranging from 12, indicating lower knowledge, to 60, indicating higher knowledge. The MAKS was not developed to function as a scale, but rather as an indicator of knowledge, with the intentional inclusion of items supported by scientific evidence to test various aspects of knowledge related to mental health [35]. In the present study, the scale exhibited a Cronbach’s alpha of 0.52, indicating reasonable internal consistency. This value is close to the value of the original scale (Cronbach’s alpha of 0.65) [35].
2. Jefferson Scale of Physician Empathy (JSPE-S) by Hojat et al. [37]; Portuguese version by Loureiro, et al. [38]—This 20-item scale uses a self-report questionnaire that assesses students’ perception of their empathetic behavior in the context of patient care. Each item has a seven-point Likert scale (1  =  Strongly Disagree, 7  =  Strongly Agree) and is grouped into three factors: (1) “perspective taking” (10 items; corresponding to the cognitive aspect of empathy); (2) “compassionate care/humaneness” (8 items, reflecting the more emotional component); (3) “standing in the patient’s shoes” (2 items; referring to the act of thinking as if one were the other person). The total score ranges from 20 to 140, with higher scores indicating higher levels of empathy. The Cronbach’s alpha of the present study was 0.80, like that of the original authors with α = 0.82 [37].
3. The Intergroup Anxiety Scale (IAS) by Stephan and Stephan [39]; Portuguese version by Querido et al. [40]—This 12-item scale assesses intergroup anxiety among nursing students, that encompass affective, cognitive, and behavioral components of anxiety. Participants were asked to evaluate their feelings regarding group relationships with individuals with mental illness, using terms such as anxious, apprehensive, comfortable, secure, worried, calm, confident, strange, tense, carefree, nervous, and at ease, on a Likert scale ranging from zero (not at all) to four (extremely). The final score is calculated by inverting the reverse-scored ratings of positive feelings and totaling all items. It can range from 0 to 48 points, with higher scores indicating higher levels of intergroup anxiety [27]. This study demonstrated good internal consistency (Cronbach’s alpha = 0.86), like the study conducted by Stephan and Stephan (Cronbach’s alpha = 0.81) [39].
4. Attribution Questionnaire (AQ-27) by Corrigan et al. [41,42] and adapted for the Portuguese population by Sousa and colleagues [43]—This questionnaire is used to assess social stigma and stigmatizing attitudes of students towards individuals with mental illness. The AQ-27 comprises a clinical vignette describing a person with a severe mental illness, such as schizophrenia, followed by 27 questions about this person. Participants rate on a scale from 1 to 9, with 1 mostly representing “no or nothing” and 9 representing “very much or completely”. Questions encompass stigma across nine dimensions, including stereotypes and discriminatory attitudes (such as Responsibility, Irritation, Dangerousness, Fear, Coercion, Segregation, and Avoidance), as well as attitudes of closeness and assistance (such as Help and Pity). Questions related to the avoidance dimension are reverse scored. Results are calculated considering the sum scores obtained for the items comprising each stereotype. Higher scores indicate a greater stigma towards individuals with mental illness, and each dimension of the AQ-27 ranges from 3 to 27 points. The Cronbach’s alpha of the present study was 0.73, slightly lower than that reported by Sousa et al. (α = 0.76) [43].

### 2.5. Intervention Program

A psychoeducational training program named “This is Me” was implemented at a nursing school to raise awareness about the stigma associated with mental illness and equip students with strategies to reduce it. In this study, the program aimed to facilitate nursing students’ interaction with individuals with mental illness, promote mental health literacy, and prevent discriminatory behaviors. This program was adapted from the “Responding to Experienced and Anticipated Discrimination” (READ) anti-stigma training, developed by INDIGO under the leadership of Professor Dr. Graham Thornicroft from King’s College London and originally designed for medical students [32]. The rationale for adapting this program is that nursing students have short clinical placements in mental health and psychiatry compared to medical students. This justifies an increase in the number of program sessions to prepare students for clinical training. However, to maintain the intervention’s quality, the core content was not changed. Investment in processes of self-knowledge and self-analysis is necessary, i.e., a person-first approach. In this sense, during the program, we highlight the personal testimony component in the curriculum because it provides a powerful and effective way for participants to understand how stigma operates in the lives of people with lived experience of a mental illness. Lastly, this study was carried out with students who developed most of their academic course during the COVID-19 pandemic period, which means that learning was limited in terms of clinical placement experiences [44]. This limitation led to adjustments in the program to better equip students with a nurturing and supportive environment to facilitate a sense of readiness in mental health and psychiatric practice.

The ambiguity inherent in the pandemic situation may have adversely affected their professional growth, educational achievements, and self-confidence, particularly in relation to fundamental nursing competencies. The adaptation of the READ program was carried out by two expert professors in mental health and psychiatric nursing, in line with the curriculum of the educational institution (the nursing degree lasts four academic years, with clinical education in Mental Health Nursing during the third year) [45]. These experts were familiar with anti-stigma interventions and followed three steps as follows: (i) needs assessment; (ii) selecting theory-informed intervention methods and practical strategies; and (iii) producing program components and materials. We used education (informational content, group discussions, and role-playing), direct contact with people with mental disorders in clinical placements, and skill development (cognitive, effective, and functional domains) as pedagogical strategies. Evidence suggests these components of intervention produce greater effectiveness in reducing stigmatization towards people with mental disorders [46,47]. Available resources and outcome variables used in previous studies were considered [48].

The program consisted of four sessions, each lasting 1.5 h. Active student participation (allowing the expression of feelings, doubts, and concerns) was encouraged in all sessions. Each session also included both a briefing and a debriefing. The first session began 24 h before the start of the Curricular Internship (CI), while the last session occurred on the final day of the CI, with intermediate sessions taking place throughout. The sessions were held biweekly over the CI’s total of nine weeks.

In the first session, the program, its objectives, and the assessment tools were introduced. The following topics were discussed: (a) Human Rights/Mental Health Rights; (b) Mental Health and Illness; (c) Stigma and Discrimination; (d) Stigma and Healthcare Professionals; (e) Stigma and Students. At the end of the session, students were asked to note any instances of discrimination or stigma they might witness in the following two weeks and share them during the second session.

The focus of the second session was on identifying, recognizing, and responding to stigmatizing situations and their consequences. Group reflection on thoughts and feelings when interacting with individuals with psychiatric disorders was conducted, addressing the following: (a) Who is affected by stigma? (b) Consequences of stigma; (c) Strategies to reduce stigma? Seven accounts of stigma were discussed and groups of four to five students were tasked with identifying a situation of discrimination or stigma they had witnessed and suggesting response strategies. This activity was conducted through case-based learning.

During the third session, students were divided into groups of four to five and asked to describe a situation of stigma and/or discrimination they observed or experienced during the Clinical Internship in Mental Health and Psychiatric Nursing. Groups then roleplayed two scenarios involving individuals with psychiatric disorders in stigmatizing situations.

The fourth session focused on reflecting, debating, and discussing action measures in the face of stigmatizing situations and how students would apply their knowledge to reduce stigma, based on the following questions: (a) What have I learned about stigma?; (b) How has the intervention changed my perception, knowledge, attitude, and approach to stigma?; (c) What will I do with this learning?

In the end, an assessment was conducted by reapplying the instruments. Additionally, an open-ended question was included about how the program contributed to self-awareness of the issue.

### 2.6. Ethical Considerations

This study adhered to the ethical principles outlined in the Helsinki Declaration and data protection guidelines, obtaining approval from the Ethics Committee of the Ribeiro Sanches School of Nursing on 19 September 2022 (approval number: P06-2022). Formal authorizations were also obtained from the authors of Portuguese versions of instruments.

Before completing the online questionnaire, participants provided their individual informed consent, responding to an initial question seeking their agreement to participate in this study. During the process, this study’s objectives and program content were explained. Furthermore, the researcher clarified that entering this study was optional and that either participating or not participating would have no effect on grades or relationships with faculty members. The questionnaires were coded with an alphanumeric number to ensure the anonymity of participants and pre/post-test intervention assessment. The benefits of participating in this study were explained, highlighting how involvement could contribute to advances in mental health and nursing practice. Students were encouraged with a certificate of participation, which they could include in their academic curricula. Furthermore, participation in the mental illness stigma reduction program for nursing students offers a range of additional benefits. These benefits include enhancing communication skills, increasing empathy, and understanding mental health issues more deeply. A space for sharing experiences and reflection was promoted, which contributed to self-awareness about stigma and how to combat it in oneself and the community. These benefits are crucial for promoting a holistic and compassionate nursing practice.

### 2.7. Data Analysis

Quantitative analyses were conducted using IBM SPSS, version 27.0 (IBM Statistical Program for Social Sciences, Armonk, New York, NY, USA). After data collection, we performed an initial analysis of all questionnaires to exclude those that were incomplete or poorly filled out. Each scale was then coded using a methodology analogous to that used by their original authors to allow for a comparison and discussion of the results. Descriptive analyses included absolute frequencies (n), relative frequencies (%), median (Md), mean (M), standard deviation (SD), range (Rg), and minimum and maximum values (Min. and Max.). Regarding inferential statistics, non-parametric statistics were employed due to the lack of normal distribution, as indicated by Kolmogorov–Smirnov tests [49]. Variables in the pre- and post-intervention groups (paired samples) were compared using the Wilcoxon test. Additionally, the Mann–Whitney U test was applied to compare independent samples whenever at least one group did not exhibit normal distribution in the studied variable. Spearman’s correlation coefficient was used to study the form and intensity of the relationship between two variables. To interpret the strength of the relationship between variables, the criterion by Marôco [49] was used: weak correlation (coefficient < 0.25), moderate (0.25 ≤ coefficient < 0.5), strong (0.5 ≤ coefficient < 0.75), and very strong (coefficient ≥ 0.75). The results of this study were considered statistically significant at a significance level of 5% [49], i.e., for *p* < 0.05.

The qualitative data were analyzed using a thematic framework approach to identify common emerging themes [50]. Two researchers (O.V. and T. C.) familiarized with qualitative data transcribed and imported the data into WebQDA software, Version 3.0 (Universidade de Aveiro, Aveiro, Portugal). An inductive technique was utilized to construct a coding scheme. If any inconsistencies in coding were detected, an agreement was reached by engaging in discussions and providing clarifications on coding categories.

## 3. Results

### 3.1. Background Characteristics of Study Participants

A total of 37 students participated in this study, representing 88.1% of those eligible (N = 42). The sample was characterized according to sociodemographic variables, as presented in Table 1.

The participants’ ages ranged from 20 to 47 years, with a mean age of 28.73 years (SD = 7.97). The participants were predominantly female (73.0%, n = 27), single (70.3%, n = 26), and Portuguese (70.3%, n = 26).

Some participants (29.7%, n = 11) reported having experienced mental illness, namely anxiety and depression. Most participants (54.1%, n = 20) reported having no family member with mental illness.

### 3.2. Outcome Analysis

#### 3.2.1. Baseline and Post-Intervention Data

The primary and secondary outcomes are shown in Table 2. The results of the assessment of attitudes of nursing students towards mental illness (primary outcome—MICA-4) are depicted in Table 2, as well as secondary outcomes: stigma-related knowledge (MAKS) intergroup anxiety (IAS), student perception regarding their empathic behavior (JSPE-S), and the attribution of social stigma and stigmatizing attitudes of students (AQ-27) (Table 2).

Overall, students showed an improvement in knowledge about stigma after the implementation of the program, resulting in less stigma. We also identified fewer stigmatizing attitudes towards people with mental illness in the post-intervention evaluation.

Wilcoxon tests comparing pre- and post-program means revealed statistically significant differences in the total MAKS (Z = −1.99, *p* < 0.05), indicating that participants improved their stigma-related knowledge (M_MAKS_Total_T0_ = 46.76 vs. M_MAKS_Total_T1_ = 49.78); in the IAS (Z = −3.42, *p* < 0.05), showing a decrease in intergroup anxiety (M_IAS_T0_ = 18.24 vs. M_IAS_T1_ = 9.76); in the stereotype of AQ27—Dangerousness (Z = −2.399, *p* < 0.05) and in AQ27—Fear (Z = −2.415, *p* < 0.05), indicating a reduction in the perception that patients were dangerous (M_Dangerousness_T0_ = 7.41 vs. M_Dangerousness_T1_ = 49.78) and a reduction in associated fear (M_Fear_T0_ = 6.43 vs. M_Fear_T1_ = 4.49); and in JSPE regarding Perspective Taking (Z = −2.555, *p* < 0.05), showing an improvement in students’ perception of their empathic behavior in the context of caring for people with mental illness, particularly in their ability to put themselves in the patient’s place (M_PerspectiveTaking_T0_ = 61.16 vs. M_PerspectiveTaking_T1_ = 65.70). The primary outcome (MICA-4) showed no significant difference post-intervention (*p* = 0.640), indicating that the anti-stigma program did not achieve the intended effect on students’ attitudes toward mental illness (Table 2).

The variables/scales (MAKS, MICA-4, IAS, AQ27, and JSPE) of students with and without contact with a family member with mental illness were compared with Mann–Whitney U tests. The results did not reveal statistically significant differences (*p* > 0.05) in any of the scales or subscales, either pre- or post-intervention.

Spearman correlations among the various scales (MAKS, MICA-4, IAS, AQ27, and JSPE) indicated statistically significant correlations between the various scales (Table 3).

A weak negative correlation between MAKS and IAS (rs = −0.380, *p* < 0.05) stands out, indicating that greater knowledge about mental illness is associated with lower intergroup anxiety. Furthermore, a moderately negative correlation was observed between JSPE and MICA−4 (rs = −0.401, *p* < 0.05), suggesting that greater empathy is associated with lower stigma. A moderately positive and significant association was also identified between JSPE and MAKS (rs = 0.479, *p* < 0.01), indicating that greater empathy is related to higher knowledge about mental illness. The AQ27 shows a positive and moderate correlation with intergroup anxiety (rs = 0.430, *p* < 0.01), indicating that higher discriminatory and stigmatizing attitudes are associated with greater anxiety.

#### 3.2.2. Program Evaluation

The participating students identified program duration and the exchange of experiences as relevant aspects of the program under study (Table 4).

##### Category 1: Relevant Aspects of the Program

Regarding the **program’s duration,** they mentioned that the “*duration was something positive, it followed the clinical teaching and allowed time to understand and assimilate*” (P9), as well as “*I think the time was sufficient, and we managed to hear each other’s ideas about stigma*” (P1).

The exchange of **experiences provided by the program**:

P4: […] *complemented the study we have been doing with the teachers and with demystifying what stigma is… it ended up being more of an exchange of ideas, on the positive side, because we were able to share some cases that have happened to us in our personal or academic lives, in this case during internships, and that will allow us to approach things differently in the future* […].

P5: *It was very important; it allowed the sharing of experiences and open discussion on the subject* […].

##### Category 2: Self-Perceived Program Outcomes

Participating students’ **self-perceived outcomes of the program** included self-awareness, reduction in fear/anxiety, reduction in prejudice, change in attitude/behavior, improvements in relationships/interaction, and recognition of stigma situations.

**Self-awareness and self-reflection** were widely mentioned by participants as a positive outcome of the program:

P12: *I suffered, in quotes, from stigma, and I didn’t know because when I went for my internship, I was afraid of not being able to interact with people and having some difficulty in communication. And it is indeed a difficulty because I think during the internship we talked about it, not knowing if we were saying the right things, always afraid of saying something inappropriate, but… they are people.*

They also provided examples of their awareness of the topic:

P15: *I think the questionnaires that the teachers gave us before and after the internship made us reflect and fostered critical reasoning. I remember a specific question, which was whether I would mind or had problems having a neighbor with a mental illness, and I remember that before the internship, when I answered that question, I thought, maybe if they were a schizophrenic* [person diagnosed with schizophrenia]*, I would mind, because, for example, if that person had an outbreak and decided to kill me, I wouldn’t like it. But after the internship and realizing that people with schizophrenia won’t kill us just because they have schizophrenia, I saw that question differently, and I realized that I indeed had a stigma.*

The **decrease in fear/anxiety** was also mentioned regularly: *I was afraid because I didn’t even know it had a stigma* (P11).

Likewise, participants described reactions associated with stigma and fear: *A gentleman approached me and started asking for help with his phone and things like that, and I caught myself thinking, unintentionally, ‘Fear, what does this crazy guy* [person with mental disease] *want?’ But then, automatically, when I thought that to myself, I thought ‘NO, this is not what you should be thinking’* (P21).

In the same vein, they identified a **prejudice reduction:**

P3: *I was afraid of not saying the right words to her, of saying something that would trigger her madness, her irritation, and her aggressiveness… With this experience, I realized that this is a prejudice and stigma that keeps us away from people with mental health problems* […].

P4: *I now know that stigma can harm people with mental illness, and I act to prevent it.*

They also **identified knowledge and its absence** as a crucial element in identifying and combating stigma:

P30: *I realized that we carry (the stigma) due to lack of knowledge, by not understanding that we actually had that prejudice about mental health. To reduce stigma, I think it’s about exchanging information, and knowledge that reduces stigma.*

P8: They concluded that: Knowledge is important to avoid prejudice.

**A change in attitude and behavior** was mentioned as a natural consequence of the previously identified categories:

P34: […] *this kind of exchange of opinions and reflection helps us be more attentive and change… as all my colleagues said, to also change our attitude towards certain situations that lead us to reflect and act more appropriately* […].

Among the results identified by the participants were also **improvements in interaction/relationship** with individuals with mental illness:

P29: *When I arrived at the internship, I had a patient with schizophrenia, and I couldn’t connect with her; I was the only one who couldn’t… I was afraid… By the end of the internship, I had built such a good relationship with her, so good… that I even miss her, and I could see how much stigma separates us from people.*

P17: *Now I can see beyond the stigma, and this allows me to establish much more meaningful and therapeutic relationships.*

Throughout the focus groups, participants reported various situations where they identified stigma and recognized learning to discern stigmatizing situations.

P11: *It helped demystify what stigma was for me, and indeed I managed to understand it and sometimes even see it happen, either during the internship or even on the street with other people who have a mental illness. Now it’s more easily identifiable, and I think it’s much easier, also for me, to demystify that stigma for other people, whether they are from the field or not, and even address the issue in the future* […].

P9: *Now I can identify the stigma better, for example when someone with depression is told that’s in your head, forget about it.*

##### Category 3: Suggestion for Improvement

As **suggestions for improvement**, they only mentioned first-person accounts of stigma situations in mental health:

P14: *My suggestion is to have someone who has experienced a mental health condition and has faced stigma, that’s what I want. I think a firsthand case would be very interesting.*

P6: *Knowing the experience of a relative of a patient who has suffered stigma can be interesting, they may be able to identify it better.*

## 4. Discussion

The present research was motivated by the scarcity of studies in Portugal investigating the impact of an awareness intervention aimed at reducing stigmatizing beliefs and attitudes among nursing students. The program “Responding to Experienced and Anticipated Discrimination (READ)” [32] that was previously tested and adapted for healthcare students emerged as a practical possibility to address, at an educational level, a perceived gap among many nursing students [36,37,38,39,40,41,42,43,44,45,46,47,48,49,50,51]. The defined structure, content, and results support its integration in the culture of the current institution. Its adoption emerged after discussing each session and adjusting the content given the cultural references of nursing students in the stage before clinical training in the specialty area.

The program’s adoption was based on recognizing the advantages of education as a strategy for reducing stigma [7,8,9,10,11,12,13,14,15,16,17,18,19,20,21,22,23,24,25,26,27,28,29,30,31,32,33,34,35,36,37,38,39,40,41,42,43,44,45,46,47,48,49,50,51,52]. This was one of the components included in the program, along with the recognition of experiential and reflective learning [53].

The implementation of the “This is Me” program resulted in the improved knowledge about stigma, reduced stigmatizing attitudes regarding danger and fear, decreased intergroup anxiety, and the improvement in the perspective-taking subscale of the empathy scale. Despite these improvements, the program did not produce significant changes in the primary outcome—attitudes towards mental illness (MICA-4).

The increase in knowledge regarding stigma and mental illness demonstrated by the participants, between the first and second assessments, highlights the need for a review of the curriculum taught in initial education because in the initial assessment, participants had only been exposed to these topics. This idea is supported by the findings of Happell et al. [54] in a systematic review conducted on attitudes toward mental illness among students in initial nursing education. They found a trend in more favorable attitudes in students who had more hours of theoretical preparation and longer clinical teaching.

The results regarding the decrease in intergroup anxiety contradict those of Querido, et al. [40], which point to the program’s cultural adaptation to specific populations in each higher education institution and to the timing of its development when these themes arise in the degree. Furthermore, regarding intergroup anxiety, it is noteworthy that a negative correlation was found between knowledge about stigma (MAKS) and intergroup anxiety. This negative correlation is consistent with other research indicating an increase in knowledge about stigma when intergroup anxiety decreases [55,56]. On the other hand, increased knowledge enhances empathy, which in turn contributes to the reduction in stigmatizing attitudes [27,55], as evidenced in this research by the correlation between empathy and knowledge (JSPE and MAKS) and the negative correlation between empathy and stigmatizing attitudes (JSE and MICA-4).

The literature suggests that other factors, such as contact with mental health professionals in mental health services, especially in the context of the relationship with clinical supervisors, may contribute to reducing stigma and stigmatizing attitudes [57,58]. Learning and sharing experiences with healthcare professionals, as well as the role of clinical supervisors in fostering reflective critical thinking, are essential in dismantling preconceived ideas among students about providing care to individuals with mental illness [59]. Contact with individuals with mental illness should occur at various stages of the illness to understand how recovery can occur. When observation of the individual’s recovery from mental illness is not possible, the perpetuation of stigma may occur [60,61].

An analysis of the responses from participating students to the question “What did you think of the intervention/program?” allowed the identification of relevant aspects of the “This is Me” program, including its duration and the exchange of experiences. The self-perceived results of the program were categorized into self-awareness, reduction in fear, decrease in prejudice, change in attitudes and behaviors, improvement in interaction/relationship, and recognition of stigma situations. Similar findings showed that an effective mental health-specific training enhanced these perceptions [62,63]. The sharing of experiences of individuals with mental illness provided participants with a deeper understanding of their concerns and the individuals themselves, while also improving interaction and relationship with them. This improvement allowed participants to develop a more genuine empathy, emotional regulation, and a fuller understanding of the experiences lived by individuals with mental illness [64]. According to Valentim et al. [65], both sharing experiences and engaging with service users, particularly within their internship context, are fundamental elements in reducing stigma. The moments of reflection and self-assessment throughout the program also contributed to understanding individuals with mental illness, promoting the consolidation of knowledge, and improving participants’ attitudes, as well as positive mental health literacy [66]. The timing and duration of the intervention (four sessions, each lasting 1.5 h) were regarded as positive. Another finding was the decrease in intergroup anxiety. When combined with improved empathy, this may indicate better attitudes and behaviors among participants [55].

Individual and collective strategies, including education and training, communication and establishing relationships with individuals who have mental illnesses, are fundamental in reducing stigma [4,14,65]. An improvement observed in their self-perception of experiencing interactions with a group of people with mental illness with less anxiety can certainly be attributed to theoretical learning, practical experience gained during clinical training (direct interaction with individuals with mental illness), as well as the experiential learning from participation in this intervention, as described in the literature on such programs [54]. Participation in the “This is Me” program facilitated the exchange of experiences among participants, which contributed to the reduction in fear and prejudice, as confirmed by other studies aimed at reducing stigma [53,54,55].

The correlation between JSPE and MAKS scores, indicating a positive relationship between stigma knowledge and empathy, highlights the importance of the program in increasing awareness about stigma, in line with the findings by Potts et al. [55]. In fact, participants mentioned their lack of knowledge about this topic before attending the program. The perceived results include self-awareness, an awareness of the topic, as well as the recognition of stigma situations. Similar results were found by other authors who evaluated anti-stigma programs [55]. The positive relationship between stigma knowledge and empathy indicates a change in students’ attitudes towards people with mental illness, as they appear to positively influence their attitudes and perceptions, although some stereotypes may persist [67].

The stigma awareness program incorporated the viewing of videos featuring public figures sharing their experiences with mental illness. This approach was highlighted in the literature as beneficial for debunking stereotypes about people with mental illness and for promoting understanding of their behavior [20,68,69]. Participants suggested that, in addition to public figures, individuals with mental illness should be included in the program to share their experiences.

The present study found no disparities in stigma and stigmatizing attitudes among individuals with and without family members with mental illness, which differs from previous findings. The literature suggests that contact with family members affected by mental illness can play a role in reducing stigma and diminishing stigmatizing attitudes [18,52].

### 4.1. Strengths and Limitations

This study is an early attempt to investigate locally the impact of an anti-stigma intervention on the attitudes and beliefs about mental illness among senior undergraduate nursing students. Subsequently, the intervention was administered by personnel from a mental health establishment. Student participation was not obligatory, enhancing the intervention’s trustworthiness and alleviating any concern that involvement would impact academic achievement. This study utilized standardized measures that demonstrated satisfactory psychometric properties in measuring the outcome variables. However, this study has some limitations that restrict the generalization of the results, including the small sample size and the variation in clinical psychiatric training contexts among participants. A follow-up three and six months after the program would have been important to assess the consolidation of acquired knowledge and the persistence of stigmatizing attitudes over time. This indicates the need for ongoing endeavors to sustain the intended outcomes over a period of time. Furthermore, this study was not pre-registered, which raises the risk of selective reporting of results. Future studies should prioritize pre-registration to enhance transparency and minimize potential biases. Another key limitation was the absence of a control group, making it difficult to attribute the observed changes exclusively to the intervention, thus limiting the validity and reliability of the findings. Likewise, future multicentric studies should be designed using experimental and control groups to look at changes due to the intervention and to establish causality by isolating the independent variable. Another potential constraint of this study is the presence of self-selection bias, which means that students who already possess fewer stigmatizing views and behaviors may be more inclined to take part in this study. Lastly, another issue may be the absence of program validation by expert consensus before implementation. This study’s application of the program and its results now contribute to that evaluation. Therefore, research with a more robust methodology is necessary to precisely evaluate the impacts of anti-stigma interventions.

### 4.2. Implications for Practice

The World Health Organization’s Mental Health Action Plan for 2013–2030 [1] has recognized addressing mental health stigma and promoting mental health as crucial goals. Future research needs to address more advanced methods of disseminating information regarding stigma and discrimination. Additionally, it is necessary to devise tactics that cater to specific groups, such as mental health service users and caregivers. Studies showed that the efficacy of various strategies varies among different subgroups of the population [21]. Research employing mixed methods must also investigate the extent of stigma across the community and see if comparable low levels are achieved, while also seeking to understand the reasons behind such findings. To promote a broader implementation, strategies should be more inclusive and incorporate peer-led participatory models [70]. This is particularly important for more vulnerable groups, who may face stigma and have unique concerns that can be better addressed through peer-led approaches.

Our study strengthens existing evidence that even brief interventions can reduce stigma and may be transferrable to real-world applications. The next steps should involve conducting a thorough analysis of the curricula used in various intervention studies, to identify the most effective elements in addressing different aspects of stigma. Additionally, collaboration with educators should be initiated to integrate stigma reduction into the standard school curriculum.

## 5. Conclusions

The “This is Me” program improved nursing students’ knowledge about stigma, reduced stigmatizing attitudes, decreased intergroup anxiety, and enhanced the ability to adopt different perspectives. The results highlight the need to review, update, or include stigma-related topics, help-seeking, and mental illness treatment in nursing curricula.

Unlike other programs, this educational program emphasized not only the reduction in stigma but also metacognition strategies including critical reflection and self-reflection. Nursing students require anti-stigma sensitization interventions to provide better care for individuals with mental illness. The anti-stigma sensitization program must include educational sessions, sharing experiences, and contact with individuals with mental illness. Reflection and self-reflection exercises have proven essential for consolidating knowledge and improving stigmatizing attitudes. This study did not achieve its expected outcomes, as no significant improvement was observed in nursing students’ attitudes toward mental illness (MICA-4). While some progress was made, such as a reduction in intergroup anxiety and increased empathy (secondary outcomes), the trial should be considered limited in terms of the desired impact.

## Figures and Tables

**Table 1:** Personal and health information of study participants (N = 37).

Personal and Health Information			
Age [Years] (Mean ± SD; Range)	28.73 ± 7.97 (20–47)		
		**n**	**%**
**Gender**	Male	10	27.0
	Female	27	73.0
	Non-binary	0	0.0
**Marital status**	Single	26	70.3
	Married/Common-law	11	29.7
	Divorced/Separated/Widowed	0	0.0
**Nationality**	Portuguese	26	70.3
	Brazilian	7	18.9
	Angolan	4	10.8
**Have you ever experienced any mental illness? ^¥^**	No	26	70.3
	Yes	11	29.7
**Do you have or have you had any contact with a family member with mental illness? ^¥¥^**	No	20	54.1
	Yes	17	45.9

^¥^ Diseases reported by students: five students reported anxiety, four depression, one epilepsy, and one Post-Traumatic Stress Disorder. ^¥¥^ Family members with mental illness: According to the degree of relationship, students reported first-degree relatives (five fathers or mothers); second and third-degree relatives (one grandmother and one great-grandmother); second-degree (four siblings), third-degree (four uncles), and fourth degree (one cousin) collateral relatives; and relatives by affinity (one mother-in-law).

**Table 2:** Change in outcome variables scores between pre- and post-intervention (N = 37).

Variables		Baseline			Post-Intervention			*Z* (Wilcoxon)	*p*
		Min–Max	M (SD)	Median (Ampl. IQR)	Min–Max	M (SD)	Median (Ampl. IQR)		
**Primary Outcome**									
**MICA-4_Total**		17–63	35.49 (8.58)	36.00 (10.00)	22–57	34.59 (8.31)	32.00 (13.50)	−0.47	0.640
**Secondary Outcomes**									
**MAKS_Total**		26–60	46.76 (6.14)	48.00 (5.00)	36–60	49.78 (4.74)	49.00 (5.00)	−1.99	**0.040**
**IAS_Total**		4–31	18.24 (7.31)	19.00 (9.50)	0–42	9.76 (9.87)	6.00 (12.00)	−3.42	**0.001**
**AQ27**	**AQ27_Total**	67–158	97.11 (18.88)	93.00 (23.00)	64–135	90.49 (17.26)	90.00 (4.00)	−1.22	0.220
	**AQ27_Responsibility**	3–18	8.30 (3.86)	8.00 (3.50)	3–15	8.70 (2.44)	9.00 (2.00)	−0.74	0.460
	**AQ27_Pity**	7–26	14.95 (5.07)	15.00 (7.00)	4–26	13.41 (6.67)	14.00 (13.00)	−0.951	0.340
	**AQ27_Irritation**	3–14	5.54 (2.83)	5.00 (4.00)	3–15	4.81 (2.97)	3.00 (2.00)	−1.356	0.180
	**AQ27_Dangerousness**	3–18	7.41 (3.69)	6.00 (5.00)	3–15	5.24 (3.04)	3.00 (4.00)	−2.399	**0.016**
	**AQ27_Fear**	3–17	6.43 (3.53)	5.00 (4.50)	3–15	4.49 (2.76)	3.00 (1.50)	−2.415	**0.016**
	**AQ27_Help**	8–27	21.86 (4.47)	22.00 (7.00)	9–27	21.84 (6.39)	24.00 (6.50)	−0.08	0.940
	**AQ27_Coercion**	5–26	14.00 (4.80)	14.00 (7.00)	3–24	13.32 (6.03)	12.00 (12.00)	−0.40	0.690
	**AQ27_Segregation**	3–19	7.38 (3.81)	7.00 (6.00)	3–15	5.57 (3.38)	4.00 (6.00)	−1.89	0.060
	**AQ27_Avoidance**	3–22	11.24 (5.42)	11.00 (7.50)	3–19	10.35 (5.31)	11.00 (8.50)	−0.73	0.470
**JSPE**	**JSPE_Total**	80–133	111.68 (15.01)	117.00 (20.00)	80–134	115.95 (16.31)	120.00 (129.50)	−0.94	0.350
	**JSPE_Perspective Taking**	44–70	61.16 (7.07)	62.00 (8.50)	49–70	65.70 (5.51)	68.00 (69.00)	−2.555	**0.011**
	**JSPE_Compassion**	8–56	42.65 (10.33)	47.00 (12.50)	8–54	42.70 (13.05)	47.00 (51.50)	−0.533	0.590
	**JSPE_Standing in Patient’s Shoes**	2–14	7.86 (2.56)	8.00 (3.50)	2–14	7.54 (3.77)	7.00 (11.00)	−0.338	0.740

Bold-face values reflect significant results.

**Table 3:** Spearman correlations between outcome variables (N = 37).

Instruments	MAKS_Total	MICA-4_Total	IAS_Total
MICA-4_Total	−0.191	-	-
IAS_Total	−0.380 *	0.180	-
AQ27_Total	−0.156	0.128	0.430 **
JSPE_Total	0.479 **	−0.401 *	−0.261

* *p* < 0.05; ** *p* < 0.01.

**Table 4:** Categories and Subcategory.

Category	Subcategory
**Relevant Aspects of the Program**	Program Duration
	Exchange of Experiences
**Self-Perceived Program Outcomes**	Self-Awareness and Self-Reflection
	Decrease in Fear/Anxiety
	Reduction in Prejudice
	Attitude/Behavior Change
	Improvements in Interaction/Relationship
	Recognition of Stigmatizing Situations
**Suggestions for Improvement**	First-Person Account

## Data Availability

The data are available upon reasonable request.

## Public Involvement Statement

There was no public involvement in any aspect of this research.

## Guidelines and Standards Statement

This manuscript was drafted against the CONSORT (Consolidated Standards of Reporting Trials) checklist for interventional studies.
